# A comparison of GenomEra® GBS PCR and GeneXpert ® GBS PCR assays with culture of GBS performed with and without broth pre-enrichment

**DOI:** 10.1007/s10096-020-03934-4

**Published:** 2020-06-13

**Authors:** S. Y. Nielsen, J. K. Møller, M. R. Khalil

**Affiliations:** 1 Department of Clinical Microbiology, Vejle Hospital, University Hospital of Southern Denmark, Odense, Denmark; 2 Department of Clinical Microbiology, Vejle Hospital, Aarhus University Hospital, Aarhus, Denmark; 3 Department of Clinical Microbiology, University Hospital of Southern Denmark, Odense, Denmark

**Keywords:** PCR, Intrapartum, Group B streptococci, *S. agalactiae*, GBS, EOGBS, Early onset, Pregnancy, Point-of-care testing

## Abstract

This study was designed to compare the performance of GeneXpert® and GenomEra® group B streptococcus (GBS) PCR assays, held up against standard culture of GBS performed with and without broth pre-enrichment. In Denmark, the strategy for preventing early onset GBS infection (EOGBS) is risk factor based. Three hundred and sixty six women fulfilling one or more of the criteria for presence of risk factors for EOGBS were prospectively included. Rectovaginal swab samples were taken intrapartum and tested bed-site by the GenomEra® and the GeneXpert® GBS PCR assays and cultured at the microbiology laboratory using Granada agar plates with and without prior growth of sampling material in selective enrichment broth. Among 366 participants tested intrapartum, 99 were GBS-positive by culture, 95 by GenomEra, and 95 by GeneXpert. Compared with culture, the GenomEra and the GeneXpert performed with a sensitivity of 91.8% and 91.7% and a specificity of 98.1% and 97.3%, respectively. A combined reference standard was established by defining true positives as either culture-positive samples or culture-negative samples where both the GeneXpert and the GenomEra GBS PCR assays were positive. Using this, the sensitivity increased to 92.2% and the specificity to 99.6% for GenomEra and to 92.0% and 96.8% for GeneXpert. The use of selective broth enrichment found only three additional GBS culture-positive samples. The performance of the two PCR methods examined was very similar and close to the findings by culture, and both PCR assays are thus applicable as rapid intrapartum bed-site tests.

## Introduction

Preventing neonatal infection with group B streptococci (GBS) still receives substantial attention from researchers worldwide aiming at the ideal identification and prevention of intrapartum transmission of GBS from mother to baby. Early-onset GBS disease (EOGBS) is still the most frequent cause of early-onset infection in neonates worldwide. More than 30% of infants delivered in the USA are now exposed to intrapartum antibiotics in order to prevent vertical transmission of GBS to the baby [1]. Internationally, the rate of EOGBS ranges from 0.23 to 3.0 per 1000 live births. In Denmark, an incidence of 0.1–0.3 per 1000 live births has been reported [2].

Colonization rates with GBS in pregnant women vary from 10 to 35% by culture of screening samples from gestational weeks 35–37 [3–5].

There are two well-known strategies for preventing EOGBS in the newborn. One is universal culture-based antenatal screening for GBS colonization in all pregnant women in gestational weeks 35–37 and treatment of all GBS positive women during labor. The other strategy is based on risk assessment alone and is used in Denmark. Here IAP is given to women with one or more of the following five risk factors: (1) previous infant with GBS infection, (2) bacteriuria during the present pregnancy, (3) a temperature > =38.0 °C during labor, (4) preterm labor < 37 gestational weeks, and (5) prelabor rupture of membranes (PROM) or preterm PROM (PPROM) ≥ 18 h. Intravenous infusion of Benzylpenicillin is recommended every 4 h for GBS prophylaxis during labor until delivery.

Despite the universal antenatal GBS screening employed in other countries, some studies report high levels of “false-negative rates,” as up to two-thirds of newborns with EOGBS are newborns of GBS antenatal screen-negative mothers [6, 7].

Rapid nucleic acid amplification tests (PCR) performed intrapartum are increasingly finding their way into clinical settings, and several studies have concluded that these screening assays perform well [8, 9].

This study was designed to compare the performance of GeneXpert® and GenomEra® GBS PCR assays, held up against the gold standard of culture of GBS using Granada agar plates with and without prior growth enrichment of the sampling material in a selective broth.

## Materials and methods

### Study population and sample collection

During the period from December 4, 2018, to July 1, 2019, 366 women were tested and enrolled prospectively at the maternity ward at Kolding Hospital, Denmark, if they fulfilled one or more of the following criteria for presence of risk factors for EOGBS: (1) bacteriuria during current pregnancy, (2) prior infant with EOGBS, (3) temperature above 38.0 °C during labor, (4) preterm labor < 37 gestational weeks, and (5) rupture of membranes ≥ 18 h.

Exclusion criteria were women younger than 18 years and women with a communication barrier.

Rectovaginal sampling was in all cases performed by midwives using two swabs simultaneously when sampling: one ESwab, Copan diagnostics, Brescia, Italy, and one of the Cepheid Sample Collection Device (Cepheid #900-0370) (the Cepheid Collection device is a dual swab device; for this study, one of the swabs was replaced by the ESwab).

The two swabs were simultaneously, held in one hand, carefully inserted into the lower one-third of the vagina and rotated to ensure uniform sampling of material on both swabs. Subsequently the two swabs were simultaneously inserted 2 cm beyond the anal sphincter and rotated to ensure uniform sampling of material from anal crypts. In all cases, sampling was performed prior to administration of IAP.

### GenomEra and GeneXpert PCR

Post sampling, the Cepheid swab was placed in the plastic transport tube of the Cepheid sample collection and immediately transferred to the designated chamber of the GeneXpert®(Cepheid Ltd., Sunnyvale, USA) GBS assay cartridge. The swab was snapped at the score mark, and the cartridge was loaded into the Cepheid GeneXpert system, for automated sample preparation and PCR. The results for specimens are reported as positive or negative based on the detection of a genomic target sequence adjacent to the *S. agalactiae cfb* gene. The total assay run time was 55 min with < 1 min of hands-on time.

The other swab was submerged into 1 ml of ESwab transport medium and immediately processed using the GenomEra® CDX system (Abacus Diagnostica, Finland), a molecular diagnostic analyzer consisting of an integrated thermal cycler and a time-resolved fluorometer.

The GenomEra GBS PCR kit targets an internal region of the cfb gene and should, based on in silico analysis of published GBS genomes and experimental data on a selection of GBS strains, be expected to detect all clinical GBS isolates (personal contact with Abacus Diagnostica, 2018). The GenomEra GBS PCR assay has only been clinically validated and CE-IVD-marked to be used with pre-enrichment broth culture of samples (GenomEra package insert).

We used a modification of the manufacturer’s instructions by applying direct swab samples instead of a 4-h pre-enrichment broth culture of samples and a sample volume of 100 μl ESwab medium instead of 10 μl of enrichment culture medium. The 100 μl ESwab medium were added to 1000 μl of the GenomEra buffer supplied for swab samples. Samples were lysed by vortexing for 5 min. From this mixture, 35 μl were transferred to the test chip and analyzed on the GenomEra® CDX system. The assay takes approximately 50 min including the final reporting of results. Results were interpreted according to the instructions of the manufacturer.

Both machines for GenomEra and GeneXpert analysis were placed at the maternity ward, and analyses were performed by trained midwives. The GenomEra assay had been implemented as the standard regime for intrapartum testing about 1 year prior to the initialization of the study.

If the primary PCR assay presented with an indecisive result (technical error), a second (repeat) test was conducted when possible. However, IAP treatment was always based on the first test result and also administered in case of a primary indecisive PCR result.

### Culture

The remaining ESwab transport medium with the swab was sent to the Department of Clinical

Microbiology, Vejle Hospital, Denmark. If received between 7 A.M and 8 P.M, the samples were cultured immediately; otherwise, they were kept at 4 °C until the next morning.

Fifty (50) microliter from the ESwab was cultured directly on Granada agar (BioMérieux) and examined after 24- and 48-h incubation under anaerobic atmosphere. Semiquantitative evaluation grading the presence of GBS as few (+), moderate (++), and numerous (+++) was performed.

Another 200 μl from the ESwab was inoculated into separate tubes with 5 ml of Todd-Hewitt broth with 1% yeast extract, 15 μg/ml nalidixic acid, and 10 μg/ml colistin (Lim broth, Becton Dickinson). These were inoculated aerobically at 37 °C overnight, and 10 μl was then subcultured on Granada agar (BioMérieux) plates and examined after 24- and 48-h incubation under anaerobic atmosphere.

B-hemolytic orange pigmented colonies from subcultures on Granada agar plates from direct plating as well as from the enrichment broth were identified as GBS by MALDI-TOF (Bruker Daltonik, Germany). GBS-like colonies that were not pigmented on Granada agar were also characterized by MALDI-TOF to identify possible non-hemolytic variants of GBS.

Culture and identification of GBS strains at the laboratory was performed by trained laboratory technicians, who were blinded for the PCR results until completion of the study period.

### Combined reference standard

A combined reference standard was established by defining true positives as either culture-positive samples or culture-negative samples where both the GeneXpert and the GenomEra GBS PCR assays were positive. Since there was absolute minimal discrepancy between culture with or without enrichment broth (three samples were only positive using the enrichment broth), these two culture methods were not evaluated separately.

### Statistics

STATA software (version 15; StataCorp LP) was used for the statistical analyses.

## Results

Among the 366 pregnant women tested intrapartum, 99 had samples that were positive by culture, 95 by GenomEra, and 95 by GeneXpert. Twenty-three assays presented with a primary undetermined test result (technical error), 2.1% (8/366) for GenomEra and 4.0% (15/366) for GeneXpert, forcing a second (repeat) test.

There were 88 samples that tested positive in both GenomEra and GeneXpert. For distribution of the results, see Table 1.

**Table 1:** Distribution of patterns of GBS test results (*N* = 366)

Culture	GenomEra PCR	GeneXpert PCR	Number
+	+	+	84
+	+	−	3
+	−	+	3
+	−	−	5
+	Error	+	1
+	+	Error	3
-	+	+	4
-	+	−	1
-	−	+	3
-	Error	−	7
-	−	Error	12
-	−	−	240

+, positive assays; −, negative assay; error, inconclusive result of primary test due to technical error

### Performance of the GenomEra and GeneXpert assays with culture of GBS as reference

Among GBS culture-positive samples, 91.8% (90/98) were positive using GenomEra, and 1.9% (5/261) of the GBS culture-negative samples were GenomEra positive. There were five false-positive and eight false-negative results, resulting in a specificity of the GenomEra PCR assay of about 98% and a sensitivity of about 92% (Table 2). The GeneXpert assay detected 91.7% (88/96) of the GBS culture-positive samples and 2.7% (7/255) of the GBS culture-negative samples.

**Table 2:** Comparison of the performance characteristics with culture as gold standard

	GenomEra GBS PCR		GeneXpert GBS PCR	
	% (*n*/*N*)	(95% CI)	% (*n*/*N*)	(95% CI)
Sensitivity	91.8 (90/98)	84.6–96.4	91.7 (88/96)	84.2–96.3
Specificity	98.1 (255/260)	95.6–99.5	97.3 (248/255)	94.4–98.9
PPV	94.7 (90/95)	88.3–97.7	92.6 (88/95)	85.8–96.3
NPV	97.0 (255/263)	94.3–98.4	96.9 (248/256)	94.1–98.4

There were seven false-positive and eight false-negative GeneXpert test results, resulting in a specificity of the GeneXpert GBS assay of about 97 % and a sensitivity of about 92% (Table 2).

For the eight false-negative PCR assays, the semiquantitative GBS culture of the samples showed that seven of these false-negative PCR results were seen in samples with only few GBS colonies by culture. Women with false-negative results by culture received IAP when the PCR result was positive in the routine GenomEra PCR assay. The amplicons from the seven false-positive PCR test results were not available for further analyses since they were discarded with the test cassettes used in the PCR assays run at the maternity ward.

### Performance of the GenomEra and GeneXpert assays with a combined standard as reference

When using the definition of the combined reference standard, 366 samples were included of which 103 were classified as true positive. Eight samples had an inconclusive result in the GenomEra assay (error) in the primary test of which one was GBS culture and GeneXpert PCR positive and therefore classified as a true GBS-positive sample. Fifteen samples had an inconclusive result (error) in the primary GeneXpert test. Three of these were GBS culture and GenomEra PCR positive and classified as true GBS-positive samples by the gold standard definition. Twelve of the samples were GBS culture and GenomEra PCR negative and classified as true negative according to the combined reference standard (Table 3).

**Table 3:** Comparison of the performance characteristics with the combined standard as reference

	Culture		GenomEra		GeneXpert	
	% (*n*/*N*)	(95% CI)	% (*n*/*N*)	(95% CI)	% (*n*/*N*)	(95% CI)
Sensitivity	96.1 (99/103)	90.0–98.5	92.2 (94/102)	85.1–96.5	92.0 (92/100)	84.4–96.5
Specificity	100 (263/263)	98.6–100.0	99.6 (255/256)	97.8–100.0	98.8 (248/251)	96.6–99.8
PPV	100 (99/99)	96.3–100.0	98.9 (94/95)	93.0–99.9	96.8 (92/95)	90.9–99.0
NPV	98.5 (263/267)	96.2–99.4	97.0 (255/263)	94.3–98.4	96.9 (248/256)	94.1–98.4

Participants were only given IAP if the routine intrapartum PCR result (GenomEra assay) came out positive. However, women with a negative GBS PCR who developed fever during labor were treated with antibiotics with broader coverage (ampicillin plus gentamicin) than penicillin according to national guidelines [10].

## Discussion

This study was designed to evaluate the diagnostic accuracy of GeneXpert® and GenomEra® GBS assays compared with standard culture including broth pre-enrichment for detection of rectovaginal carriage of group B streptococci using direct sample material from a combined vaginal/rectal swab.

Compared with culture, the sensitivities for GBS detection by GenomEra and GeneXpert were very similar: 91.8% and 91.7%, respectively (Table 2). Using the combined standard as reference, the sensitivity increased to 92.2% and 92.0%, respectively. The sensitivity of culture was 96.1. The difference in sensitivity between the three GBS assays was not statistically significant as were the specificities (Table 3).

Several studies comparing GeneXpert PCR with standard culture have been performed and have shown a sensitivity of 85.7% [11], 89% [12], 98.5% [8], 86.7% [13], and 100% [14] for GeneXpert. One recent study compared BD MAX GBS and GenomEra GBS assays and found a sensitivity of 79.1% for GenomEra and no statistical differences between the performance of culture, BD MAX, and GenomEra [15].

In a recent study, we compared the diagnostic accuracy of BD MAX™ GBS and GenomEra® GBS assays for a rapid intrapartum PCR detection of vaginal carriage of group B streptococci based on frozen samples analyzed retrospectively. Here we found that BD MAX™ GBS had a slightly better sensitivity but lower specificity compared with GenomEra GBS, although the differences in performance were not statistically significant. Both PCR assays did not detect GBS in all culture-positive vaginal samples but detected on the other hand GBS in several culture-negative specimens. In that study [15], the sensitivities of the three assays (culture, BD MAX, and GenomEra) were 83.0%, 87.3%, and 79.1% compared with a combined reference standard defined as we have done in this study. The present study using intrapartum PCR assays on fresh samples thus corroborates the findings of the former study showing that GBS PCR assays are comparable to culture of GBS from rectovaginal samples.

We found a number of samples with primary invalid results/errors: 8 for the GenomEra and 15 for GeneXpert. This corresponds to an invalid results rate of GenomEra of about 2% which is in accordance with our previous study including the GenomEra GBS assay. The GeneXpert assay had almost twice the invalid results rate.

In a recent Danish study, analysis of the swabs was solely performed at the Department of Clinical Microbiology by trained lab technicians instead of testing at the labor ward, resulting in only one of 106 GeneXpert PCR results(< 1%) being invalid [14].

When performed in a labor ward setting, fluctuating invalid rates have been reported for GeneXpert; Mueller et al. reported 55.3 % initially invalid GeneXpert results, reduced to 13.4% after 2-h training of midwives [11]. Håkansson et al. reported 15% invalid results as the midwives’ experience improved [16], and Helali et al. had 9% invalid results [17].

Not all hospitals have laboratory facilities right next to the labor ward or accessible trained lab personnel on a 24-h basis, and we decided to perform our study in a clinical setting representative for many hospitals/labor wards.

We trust that the routine experience in our labor ward with of one GBS PCR assay before the study has had a positive effect of the handling of both of the PCR analyses in our study.

The number of erroneous results is an important criterion when assessing the feasibility of this point-of-care technology. The therapeutic implication of an invalid test result may very well be instituting IAP since there will often be no time to wait for a rerun in a clinical setting with women in labor.

Among strengths of our study is the fact that the vaginal swabs are prospectively collected from women fulfilling one or more of the risk factor criteria and also that the two PCRs and the two versions of GBS culture (with and without broth pre-enrichment) were tested on the same set of samples, excluding sampling bias. Another strength is that all analyses were performed on fresh specimens immediately after sampling, avoiding freeze-thawing cycles on kept material.

Nabil A El Aila et al. [18] found that the difference in the detecting rates between the direct plating of the rectovaginal swab on the Granada medium and plating after prior Lim broth enrichment is only 4%. Our study, with just three samples (one negative in both PCR’s, two positive in both PCR’s) that were only culture positive after broth enrichment corresponding to about 3% (3/99), emphasizes that this broth pre-enrichment step is of limited value in a clinical setting.

Approximately 5–8% of GBS are non-hemolytic because they lack a beta-hemolysin [19]. It may also be a strength in this study that we, in order to address this, performed MALDI-TOF identification on all non-orange GBS-like colonies on the Granada agar culture plates. No GBS isolates were identified, whereas many *Enterococcus* and coagulase-negative staphylococci were found.

A weakness of the study is the potential bias introduced by the different handling of the two PCR assays and the prior experience of the midwives in performing the assays. Due to the pipetting steps, the GenomEra analysis is technically slightly more challenging than the GeneXpert. However, the midwives had experience in using the GenomEra assay as it had been implemented as routine testing 14 months prior to the study.

Intrapartum PCR assay performs better than the antepartum culture for identification of GBS vaginal carriers during labor [20]. PCR was found to be both cost-effective and applicable as rapid intrapartum bed-site tests. Studying the risk-based approach in combination with a PCR assay in detecting vaginal carriage of GBS in laboring women at term shows that in programs that aim to treat all laboring women with vaginal GBS colonization with penicillin, the PCR-GBS will perform well (sensitivity 83% and specificity 97%). In programs aiming to treat only GBS carriers among those with risk factors of EOGBS, a reduction of penicillin usage by two-thirds may be possible [21]. Regardless of the strategy used, methods with more rapid and accurate identification of GBS carriers at labor will better identify women at risk for GBS neonatal transmission and thereby optimize the use of IAP and reduce the incidence of EOGBS. El Helali et al. performed a large cost-effectiveness study of intrapartum PCR versus antenatal culture. They reported a significant decrease in the proportion of EOGBS in the intrapartum PCR negative group from 0.36 to 0.04% as well as a significant reduction in the use of antibiotics [22].

As of today, the discussion of intrapartum PCR versus antenatal culture is still ongoing, as is the challenge of testing and treating the right women. Nanduri et al. found that among the mothers of 1277 infants with EOGBS, 48% had no indications for IAP and did not receive it and 22 % failed to receive IAP despite having indications [1].

Our study evaluates intrapartum testing, and we find that the results of the GenomEra and the GeneXpert PCR assays were very similar and close to culture with regard to sensitivity and specificity. Both PCR methods tested are thus applicable in a clinical setting.

## Data Availability

Data archiving is not mandated but data will be made available on reasonable request.
